# A Highly Sensitive and Specific Detection Method for *Mycobacterium tuberculosis* Fluoroquinolone Resistance Mutations Utilizing the CRISPR-Cas13a System

**DOI:** 10.3389/fmicb.2022.847373

**Published:** 2022-05-13

**Authors:** Xiaopeng Bai, Panqi Gao, Keli Qian, Jiandong Yang, Haijun Deng, Tiwei Fu, Yuan Hu, Miaomiao Han, Huizhi Zheng, Xiaoxia Cao, Yuliang Liu, Yaoqin Lu, Ailong Huang, Quanxin Long

**Affiliations:** ^1^Key Laboratory of Molecular Biology on Infectious Diseases, Ministry of Education, Chongqing Medical University, Chongqing, China; ^2^Department of Infection Control, The First Affiliated Hospital of Chongqing Medical University, Chongqing, China; ^3^Urumqi Municipal Centre for Disease Control and Prevention, Xinjiang, China; ^4^Chongqing Key Laboratory for Oral Diseases and Biomedical Sciences, Chongqing Medical University Stomatology College, Chongqing, China

**Keywords:** CRISPR-cas13a, crRNA screening, *Mycobacterium tuberculosis*, fluoroquinolone resistance mutations, single-base resolution DNA detection

## Abstract

**Objectives:**

CRISPR-Cas13a system-based nucleic acid detection methods are reported to have rapid and sensitive DNA detection. However, the screening strategy for crRNAs that enables CRISPR-Cas13a single-base resolution DNA detection of human pathogens remains unclear.

**Methods:**

A combined rational design and target mutation-anchoring CRISPR RNA (crRNA) screening strategy was proposed.

**Results:**

A set of crRNAs was found to enable the CRISPR-Cas13 system to dramatically distinguish fluroquinolone resistance mutations in clinically isolated *Mycobacterium tuberculosis* strains from the highly homologous wild type, with a signal ratio ranging from 8.29 to 38.22 in different mutation sites. For the evaluation of clinical performance using genomic DNA from clinically isolated *M. tuberculosis*, the specificity and sensitivity were 100 and 91.4%, respectively, compared with culture-based phenotypic assays.

**Conclusion:**

These results demonstrated that the CRISPR-Cas13a system has potential for use in single nucleotide polymorphism (SNP) detection after tuning crRNAs. We believe this crRNA screening strategy will be used extensively for early drug resistance monitoring and guidance for clinical treatment.

## Introduction

Tuberculosis (TB) is still a serious public health concern. TB is usually caused by *Mycobacterium tuberculosis* (*M. tuberculosis*). Approximately one-quarter of the world’s population has been infected with *M. tuberculosis* (World Health Organization [Who], 2020). In 2019, TB was the leading cause of death worldwide from a single infectious agent, except for SARS-CoV-2 infection (World Health Organization [Who], 2020). The newest guidelines published by the World Health Organization (WHO) confirmed their recommendations for drug susceptibility testing (DST) at the start of therapy for all previously treated patients to improve the very poor outcomes of patients who have a high possibility of multiple drug-resistant *M. tuberculosis* (MDR-TB) infection. Recent studies have attempted to improve clinical outcomes and shorten the duration of MDR-TB treatment by adding new drugs or further classifying MDR-TB, such as identification of “simple MDR-TB” (only resistant to rifampin and isoniazid but susceptible to other second-line drugs) (Li et al., 2019). The newly approved antibiotic bedaquiline-containing STREAM trial is now in stage 2 and is expected to shorten the course to 28 weeks (Moodley et al., 2016). Furthermore, the WHO recommended a standardized shorter regimen (Bangladesh regimen) for MDR-TB patients who are probably susceptible to second-line drugs (Van Deun et al., 2010), which shortens the treatment regimen to 9 months with excellent successful outcomes (87.9%, 95% CI, 82.7–91.6). The Bangladesh regimens contain fluoroquinolone (ofloxacin or gatifloxacin), kanamycin, and prothionamide as the core drugs, and the initial resistance to fluoroquinolones is related to the therapy failure rate. Performing molecular DST of fluoroquinolones or pyrazinamide has been recommended before the selection regimen to ensure the successful application of shortened MDR-TB regimens. However, the traditional phenotypic drug susceptibility test requires weeks to detect resistance due to the extremely slow growth of *M. tuberculosis* (Van Deun and Chiang, 2017; Sun et al., 2019). Therefore, it is urgent for us to develop a rapid, sensitive and specific detection method for antibiotic resistance mutations in *M. tuberculosis*.

Prokaryotic adaptive immune systems use CRISPRs (clustered regularly interspaced short palindromic repeats) and CRISPR-associated (Cas) proteins to cleave foreign genetic elements (Mohanraju et al., 2016; Wright et al., 2016). CRISPR-Cas systems have been widely used to edit genomes, track molecules in live cells, and decrease or increase gene expression *in vivo* in different organisms (Barrangou and Doudna, 2016; Pickar-Oliver and Gersbach, 2019). The CRISPR-Cas13 system belongs to the type VI CRISPR-Cas system and includes a single Cas protein. The Cas13 protein possesses two enzymatically distinct ribonuclease activities: one RNase activity is responsible for pre-crRNA processing, while the other RNase activity, provided by the two HEPN (higher eukaryotes and prokaryotes nucleotide-binding) domains, is required for the degradation of target RNA during viral interference (East-Seletsky et al., 2016; Konermann et al., 2018; Yan et al., 2018). Once the Cas13a-crRNA complex assembles with the target RNA, the HEPN domain will be activated and present general RNase activity (Shmakov et al., 2015; East-Seletsky et al., 2016). Several molecular diagnostic methods that are rapid, accurate and sensitive have been developed based on the non-specific RNase of activated Cas13a (Gootenberg et al., 2017; Shan et al., 2019).

Although dozens of studies have employed the CRISPR-Cas13a system in nucleic acid detection, including the rapid detection of SARS-CoV-2 infection (Gootenberg et al., 2017; Barnes et al., 2020; Joung et al., 2020; Wang et al., 2020; Fozouni et al., 2021), there is still a lack of information for crRNA design in the application of the Cas13a system in single nucleotide polymorphism (SNP) identification. Introducing synthetic mismatches into the spacer region of crRNA is the most commonly used strategy to distinguish SNPs in CRISPR-Cas13a systems (Gootenberg et al., 2018; Myhrvold et al., 2018; Shan et al., 2019; Zhou et al., 2020). However, the locations of synthetic mismatches in crRNA are uncertain, and the underlying enhancing mechanism remains unclear. Here, we proposed a combined rational design and target mutation-anchoring screening strategy that improved the specificity of CRISPR-Cas13a systems. The panel of crRNAs presented here can efficiently discriminate fluoroquinolone resistance mutations in *M. tuberculosis*.

## Materials and Methods

### Sample Collection and Human Subjects Approvals

The study collected 75 *M. tuberculosis* strains, including 35 fluoroquinolone-resistant and 40 fluoroquinolone-sensitive strains, which were collected from the Urumqi Municipal Centre for Disease Control and Prevention. The study was approved by the Ethics Committees of Urumqi Municipal Centre for Disease Control and Prevention. Subjects from Urumqi Municipal Centre for Disease Control and Prevention provided written consent to participate in the study, including consent for the storage of *M. tuberculosis* strains isolated from sputum samples for future testing.

### LwCas13a Protein Purification

The pC013-Twinstrep-SUMO-huLwCas13a plasmid was a gift from Feng Zhang (Addgene plasmid # 90097).^1^ Briefly, the pC013-Twinstrep-SUMO-huLwCas13a plasmids were transformed into *Escherichia. coli* BL21 (DE3), protein expression was induced with 0.5 mM IPTG (Sangon Biotech), at 18°C for 16 h. Cell pellet was re-suspended in lysis buffer (20 mM Tris-HCl, 500 mM NaCl, 1 mM DTT, pH 8.0) supplemented with protease inhibitors (Ultra EDTA-free tablets), lysozyme, and benzonase followed by sonication. Lysate was cleared by centrifugation and filtered through a Stericup 0.22 μm filter (Millipore). Filtered supernatant was applied to StrepTactin Sepharose (IBA) and incubated with rotation for 2 h followed by washing of the protein-bound StrepTactin resin three times in lysis buffer. The resin was re-suspended in SUMO digest buffer (30 mM Tris-HCl, 500 mM NaCl 1 mM DTT, 0.15% Igepal (NP-40), pH 8.0) along with 100 μL (10 U/μL) of SUMO protease (Hai gene) and incubated overnight at 4°C with rotation. The protein eluate was isolated by spinning the resin down. The supernatant was concentrated *via* a Centrifugal Filter Unit to 1 mL in S200 buffer (10 mM HEPES, 1M NaCl, 5 mM MgCl_2_, 2 mM DTT, pH 7.0), and was further loaded onto a gel filtration column (Superdex^®^ 200 Increase 10/300 GL, GE Healthcare Life Sciences) *via* fast protein liquid chromatography (FPLC). The resulting fractions from gel filtration were analyzed by SDS-PAGE and fractions containing LwCas13a were pooled and buffer exchanged into Storage Buffer (600 mM NaCl, 50 mM Tris-HCl, 5% glycerol, 2 mM DTT, pH 7.5) and frozen at −80°C for storage.

### ssRNA and crRNA Preparation

Partial sequence of quinolone resistance-determining region (QRDR) in the *gyrA* gene of *M. tuberculosis* H37Rv was obtained by synthesis (Tsingke Biotechnology) (sequence listed in Supplementary Table 1). A variety of QRDR plasmids containing fluoroquinolone resistance related mutations were obtained using Fast Site-Directed Mutagenesis Kit (TIANGEN, KM101). Nucleic acid targets were amplified with 2 × Taq DNA Polymerase (Invitrogen) *via* PCR with primers appended T7 promoter. Purified dsDNAs was incubated with T7 polymerase overnight at 37°C using the HiScribe T7 High Yield RNA Synthesis kit (New England Biolabs). Transcription reactions were quenched to reduce template DNA *via* treatment with DNase I (TAKARA) at 37°C for 15 min and then ssRNAs subsequently purified with the HiPure RNA Pure Micro Kit (Magen).

For preparation of crRNAs, ssDNA (Tsingke Biotechnology) (sequence listed in Supplementary Table 2) complementary to the crRNA sequence was ordered with an appended T7 promoter sequence. crRNA DNA was annealed to a short T7 primer (final concentrations 10 μM) to produce a partial dsDNA to increase T7 RNA polymerase transcription efficiency. The process of partial dsDNA as template transcribing into RNA was same as mentioned above.

### LwCas13a Collateral Detection

Detection assays were performed with 450 nM purified LwCas13a, 900 nM crRNA, 120 nM quenched fluorescent RNA reporter (RNAse Alert v2, Thermo Scientific), 0.5 μL murine RNase inhibitor (New England Biolabs), 100 ng of background total human RNA (purified from HEK293FT culture), and 1,800 nM of input nucleic acid target in nuclease assay buffer (40 mM Tris-HCl, 60 mM NaCl, 6 mM MgCl_2_, pH 7.3) to 50 μL final volume. Reactions were incubated in a fluorescence plate reader (BioTek) for up to 60 min at 37°C with fluorescence measurements taken every 1 min (λex: 490 nm; λem: 520 nm).

The integrated reaction combining T7 RNA polymerase transcription and LwCas13a collateral detection consisted of 450 nM LwCas13a, 900 nM crRNA, 120 nM quenched fluorescent RNA reporter (RNAse Alert v2, Thermo Scientific), 0.5 μL murine RNase inhibitor (New England Biolabs), 100 ng of background total human RNA (purified from HEK293FT culture), and increasing amounts of DNA input, 0.5 μL T7 RNA polymerase Mix (New England Biolabs), 2.5 μL NTP Buffer Mix (New England Biolabs, E2050S) in nuclease assay buffer (40 mM Tris-HCl, 60 mM NaCl, 6 mM MgCl_2_, pH 7.3) to 50 μL final volume. Reactions were allowed to proceed for 80 min at 37°C on a fluorescent plate reader (BioTek) with fluorescent kinetics measured every 1 min (λex: 490 nm; λem: 520 nm).

### Phenotypic Drug-Susceptibility Testing

Indirect drug-susceptibility testing was performed from the positive *M. tuberculosis* culture with the use of the proportion method. The critical concentrations used for each drug were 4 μg per milliliter for ofloxacin, 1 μg per milliliter for moxifloxacin, 2 μg per milliliter for levofloxacin. Briefly, prepare 1 mg/ml bacillary suspension (McFarland No. 1), further diluted in sterile distilled water to give 10^–2^ and 10^–4^ dilution. Drug containing media and plain control median were inoculated with 1 loop-full bacillary suspension in indicated dilution. The slopes were incubated at 37^°^C for 4 weeks. Resistance proportion (clones in drug containing medium/clones in control medium) > 1% were considered as resistance to certain drug. Para-nitrobenzoic acid (PNB) and thiophen-2-carboxylic acid hydrazide (TCH) growth tests were further done to rule out the non-tuberculous mycobacteria (NTM). Drug containing medium were provided by BASO diagnostics, Inc., Zhuhai, China.

### DNA Sequencing

Partial sequence of quinolone resistance-determining region (QRDR) in the *gyrA* of *Mycobacterium tuberculosis* H37Rv was 300 nt (7,431–7,730) and synthesized by Tsingke Biotechnology. Primers (T7F and T7R) for amplification of plasmids or clinical specimens including this region used in the study were shown in Supplementary Table 1. Primers for constructing template plasmids related to fluoroquinolone tolerance of *M. tuberculosis* were synthesized by Tsingke Biotechnology (sequence listed in Supplementary Table 1). ssDNA templates for T7 RNA polymerase *in vitro* transcription to produce crRNA and all primers in this study were with PAGE purification.

### Statistic

Fluorescence intensity and signal ratio were described as mean ± SD. Significant differences between mutation and wild type were evaluated by *t*-test. *P*-value less than 0.05 was considered statistically significant, *P*-values less than 0.001, 0.01, and 0.05 were shown as ^***^, ^**^, and *, respectively. The bar plots show the means ± SD. Receiver Operator Characteristic (ROC) curves was generated by *pROC* package, and the area under the curve (AUC) was estimated for quantifying accuracy of the detection of fluoroquinolones resistance mutation in clinical samples. All statistical analyses were performed using R software, version 3.6.0.

## Results

### *Mycobacterium tuberculosis* Fluoroquinolone Resistance-Related Mutations

Based on our previous epidemiological survey of fluoroquinolone resistance in *M. tuberculosis* from China and other tuberculosis drug resistance mutation databases (Sandgren et al., 2009; Long et al., 2012; Rosenthal et al., 2017; Dai et al., 2019), we summarized the high-frequency mutations in *gyrA* that were related to fluoroquinolone resistance and designated these mutations as targets of CRISPR-Cas13a crRNA screening.

### Setup of CRISPR-Cas13a Trans-Cleavage Activity Systems for crRNA Screening

**FIGURE 1:**
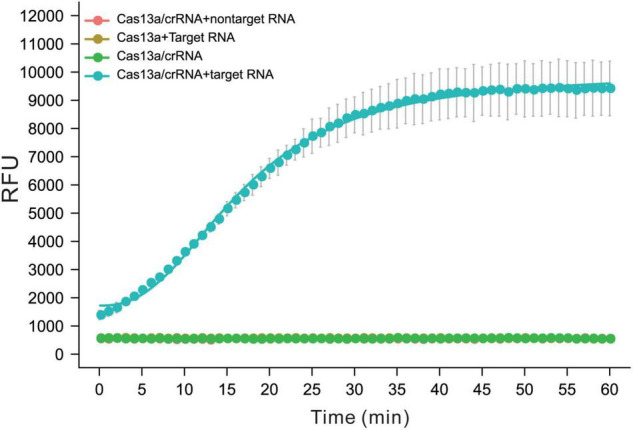
The performance of Cas13a/crRNA for detection of ssRNA was transcript from M.tb *gyrA*. The activity of trans-activated Cas13a RNase was detected by quenched fluorescent RNA reporter (RNase Alert V2, Thermo Scientific). Reactions were processed at 37^°^C on a fluorescent plate reader (BioTek) with fluorescent signal measured every 1 min (*n* = 3 replicates, bars represent means ± SD).

The purity of the LwCas13a protein was analyzed by sodium dodecyl sulfate-polyacrylamide gel electrophoresis (PAGE) and Coomassie blue staining. Then, the crRNA-guided target-activated trans-endonuclease activity of LwCas13a was determined using crRNA containing a 28 nt spacer and target RNA by fluorescence assay (Figure 1). ssRNA transcribed from a plasmid containing a *gyrA* fragment (188 nt 7,462–7,649) was chosen as the model target, and crRNA with a 28 nt spacer that was completely complementary to *gyrA* (7,462–7,649) was used to verify the specificity of the CRISPR-Cas13a/crRNA system. Another 188 nt ssRNA transcript was used as the non-target control (sequences listed in Supplementary Table 1). As shown in Figure 1, the fluorescence intensity of the sample with the Cas13a/crRNA complex and *gyrA* target ssRNA increased rapidly and reached saturation within 40 min. However, the fluorescent signal barely changed without crRNA or target ssRNA. The non-specific ssRNA also could not induce fluorescence enhancement, confirming that CRISPR-Cas13a crRNA systems can only be activated by specific ssRNA.

### Rational Design Spacers Enable CRISPR-Cas13a to Have Single-Base Specificity

**FIGURE 2:**
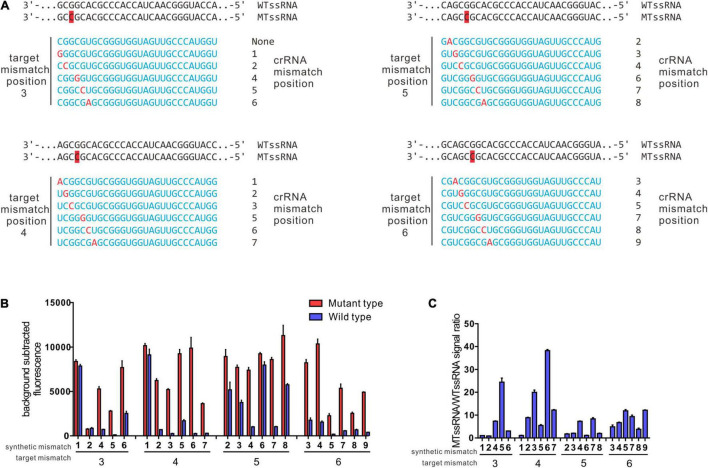
Identification of ideal synthetic mismatch position relative to mutation in the target sequence by rational design. **(A)** Spacer sequences for evaluation of ideal synthetic mismatch position to detect a mutation between wt ssRNA and mt ssRNA. Target mismatch was paced at 3, 4, 5, 6, and synthetic mismatches in red were shifted in position relative to target mismatch as presented. **(B)** Collateral cleavage activity of crRNA on wt ssRNA or mutant RNA at different position. There were four sets of crRNAs that containing target mismatch at either position 3, 4, 5, or 6 with the spacer region (*n* = 3 technical replicates; bar represent mean ± SD). **(C)** Specificity ratios of crRNAs in B. Specificity ratios are calculated as the ratio of the mutant ssRNA (on target) collateral cleavage to the wildtype ssRNA (off target) collateral cleavage. (*n* = 3 technical replicates, bar represent mean ± SD).

Previous studies showed that LwCas13a target cleavage was reduced when there were two or more mismatches in the target crRNA duplex but was relatively unaffected by single mismatches (Gootenberg et al., 2017). On a target with a mutation at the third position, LwCas13a shows maximal specificity when the synthetic mismatch is in position 5 of the spacer and the spacer length is 28 nt (designated 3–5crRNA hereafter) (Gootenberg et al., 2017). Based on this approach, taking GyrA G88A as the target mutation, we also shifted the target mutation across positions 3–6 and tiled synthetic mismatches in the spacer around the mutation (Figure 2A). In our data, the target mutation at position 4 of the spacer and synthetic mismatch at position 6 (4–6 crRNA) showed the highest specificity ratio (38.22 ± 0.91) with a comparable high fluorescence intensity (9891.34 ± 2095.38) (Figures 2B,C). The 3–5 crRNA design strategy is the suboptimal strategy in G88A SNP recognition with a specificity ratio of 24.45 ± 3.09.

**FIGURE 3:**
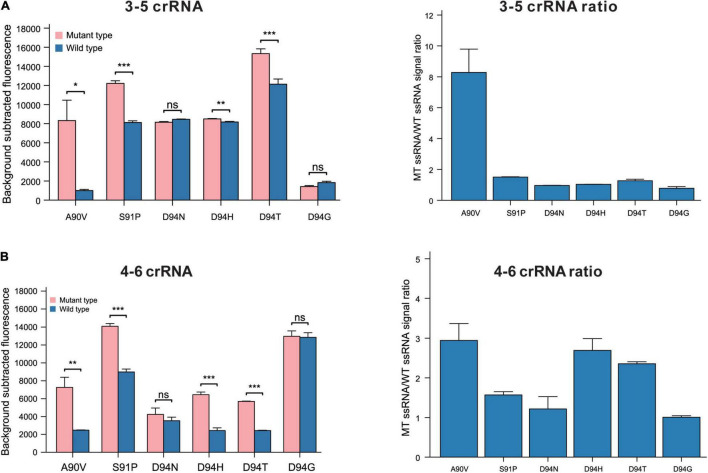
The performance of 3–5 and 4–6 crRNA design strategy in recognition of SNPs leads to aa substitutions in GyrA 90, 91, and 94. **(A)** Performance of rational designed 3–5 crRNAs in different SNPs recognitions (*n* = 3 technical replicates; bars represent mean ± SD). **(B)** Performance of rational designed 4–6 crRNAs in different SNPs recognitions (*n* = 3 technical replicates; bars represent mean ± SD). ***p < 0.001; **p < 0.05; ns, not significant.

Based on the performance of the 4–6 and 3–5 crRNA strategies in recognizing SNPs leading to the G88A, we evaluated the 4–6 and 3–5 crRNA design strategies in recognizing SNPs leading to A90V, S91P, and D94N (H)(T)(G). The 3–5 crRNA can effectively distinguish SNPs leading to aa substitutions in A90V, with a specificity ratio of 8.29 ± 1.51 (Figures 3A,B). The function of crRNAs based on this rational design strategy in the recognition of SNPs leading to S91P or D94N (H)(T)(G) is undesirability (S91P substitution, 3–5 crRNA:1.50 ± 0.02, 4–6 crRNA:1.57 ± 0.08, D94N, 3–5 crRNA:0.96 ± 0.01, 4–6 crRNA: 1.22 ± 0.31, D94H, 3–5 crRNA:1.04 ± 0.01, 4–6 crRNA: 2.69 ± 0.3, D94T, 3–5 crRNA:1.27 ± 0.1, 4–6 crRNA:2.36 ± 0.05, D94G 3–5 crRNA:0.78 ± 0.11, 4–6 crRNA:1.01 ± 0.04). These data demonstrated that the target mutation at the 5’ region (position 3–6) combined with synthetic mismatches around the mutation does not fit all strategies in the design of crRNAs that have single-base specificity.

### Target Mutation-Anchored Screening Strategy Enables CRISPR-Cas13a to Have Single-Base Specificity

**FIGURE 4:**
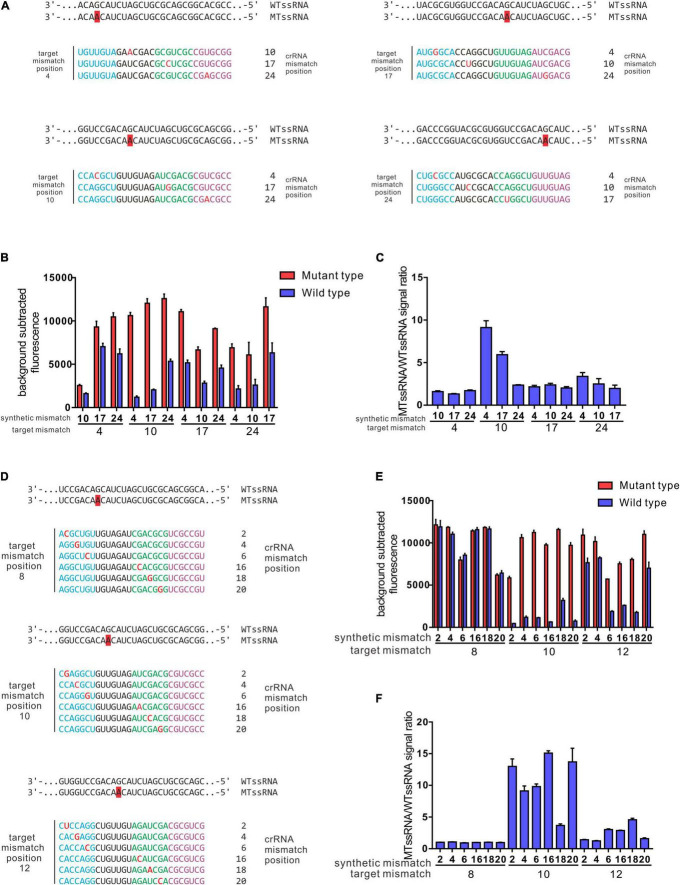
Target mutation anchored screening strategy enables a robust resolving power of SNPs led GyrA D94N aa substitution. **(A)** Depiction of crRNA design strategy for the first-round screening. **(B)** Collateral cleavage activity of crRNAs from first-round screening on wt ssRNA or mutant RNA at different positions. **(C)** Specificity ratios are calculated as the ratio of the mutant ssRNA (on target) collateral cleavage to the wildtype ssRNA (off target) collateral cleavage. crRNAs that contain target mutation were anchored in region B, while the synthetic mismatch introduced in regions A and C was higher than in other combinations. **(D)** Depiction of crRNAs generated from fine-tuning for position combinations of target mismatch and synthetic mismatch. **(E)** Collateral cleavage activity of crRNAs from fine-tuning process on wt ssRNA or mutant RNA. **(F)** Specificity ratios of crRNAs after fine-tuning on wt ssRNA and on mutant ssRNA. The performance of 3–5 and 4–6 crRNA design strategy in recognition of SNPs leads to aa substitutions in GyrA 90, 91, and 94. **(A)** Performance of Rational designed 3–5 crRNAs in different SNPs recognitions (*n* = 3 technical replicates; bars represent mean ± SD). **(B)** Performance of rational designed 4–6 crRNAs in different SNPs recognitions (*n* = 3 technical replicates; bars represent mean ± SD). ***p < 0.001; **p < 0.05; ns, not significant.

**FIGURE 5:**
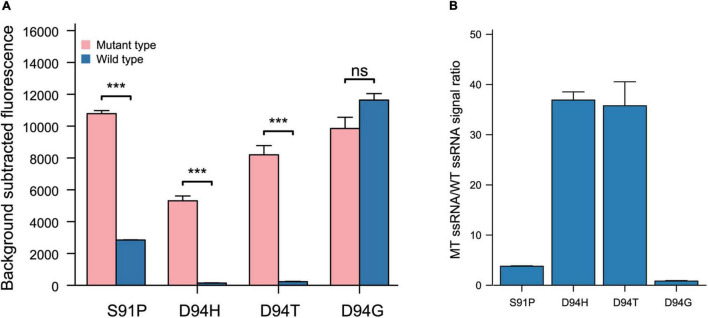
The performance of 10 16 design strategy from target mutation anchored screening strategy resolving power of SNPs lead S91P, D94H, D94T, and D94G. **(A)** Performance of target mutation anchored screening strategy 10–16 crRNAs in different SNPs recognitions (*n* = 3 technical replicates; bars represent mean ± SD). **(B)** Performance of target mutation anchored screening strategy 10–16 crRNAs in different SNPs recognitions (*n* = 3 technical replicates; bars represent mean ± SD). ***p < 0.001; ns, not significant.

Previously, data showed that a target mutation at the 5′ region (position 3–6) combined with synthetic mismatches around the mutation did not give a robust ability to distinguish certain SNPs (Figures 3A,B). In further studies, the 28 nt spacer was divided into 4 regions (region A: 1–7 nt, region B: 8–14 nt, region C: 15–21 nt, and region D: 22–28 nt); target mutations were introduced into regions A, B, C and D; and synthetic mismatches were introduced into the middle of the other 3 regions (Figure 4A). The optimal target mutation was identified after the first round of screening, the optimal position combination of the target mutation and synthetic mismatch was settled, and the fine tuning of synthetic mismatches was further carried out in the second round of screening. Based on this screening strategy, we designed crRNAs that targeted SNPs leading to GyrA D94N substitution, and the target mutation was anchored in region B, while the synthetic mismatches introduced into regions A, C and D, with specificity ratios of 9.12 ± 1.37, 5.93 ± 0.63 and 2.35 ± 0.08, were obviously higher than those of other mismatch combinations (Figures 4B,C). During fine tuning, target mismatches were further set to positions 8, 10, and 12 in region B, while synthetic mismatches were introduced into positions 2, 4 and 6 in region A and positions 16, 18, and 20 in region C (Figures 4D–F). When the target mismatch was set to position 10 and the synthetic mismatch to position 16 (crRNA 10–16), the strongest specificity ratio (15.09 ± 0.64) was found, and that for the first round of screening was also higher (crRNA 10–4 9.12 ± 1.37). For SNPs leading to D94H and D94T substitutions, which were in the same position as D94N, crRNA 10–16 presented an excellent specificity ratio (D94H 36.90 ± 1.63, D94T 35.77 ± 4.78) (Figures 5A,B). Notably, the 10–16 design strategy could not obtain a crRNA with a desirable specificity ratio for discriminating the D94G aa substitution, even though the target mutation leading to D94G was just one nucleotide shift compared with D94N, H, and T (Table 1 and Figures 5A,B). Thus, the single-base specificity crRNA is highly sensitive to the target, and no general rule can be directly applied to other conditions. Based on this Target Mutation-Anchored Screening Strategy (TMAS) strategy, we further obtained crRNAs that can efficiently distinguish SNPs that lead to S91P aa and D94G aa substitutions from wild-type SNPs with signal ratios of 11.98 ± 0.21 and 9.29 ± 0.73, respectively (Supplementary Figures 1A–F, 2A–F).

**TABLE 1:** Mutations associated with resistance to fluoroquinolones in *Mycobacterium tuberculosis*.

Amino acid in GyrA	Substitutions in GyrA	nt* mutations in *gyrA*
G88A	Gly-Ala	GGC-GCC
A90V	Ala-Val	GCG-GTG
S91P	Ser-Pro	TCG-CCG
D94N	Asp-Asn	GAC-AAC
D94H	Asp-His	GAC-CAC
D94T	Asp-Tyr	GAC-TAC
D94G	Asp-Gly	GAC-GGC

*nt, nucleotide acid.

Overall, based on the rational design and TMAS strategy, we screened a set of crRNAs with desirable fluoroquinolone resistance mutation-resolving ability (G88A, 4–6 crRNA, 38.22 ± 0.91; A90V, 3–5 crRNA, 8.29 ± 1.51; S91P, 10–17 crRNA, 11.98 ± 0.21; D94N, 10–16 crRNA, 15.09 ± 0.64; D94H, crRNA 10–16, 36.90 ± 1.63; D94T, 10–16 crRNA, 35.77 ± 4.78; and D94G, 17–10 crRNA, 9.29 ± 0.73).

### Analyzing the Performance of crRNA-Guided LwCas13a Systems for the Detection of Fluoroquinolone Resistance Mutations in Clinical Samples

**FIGURE 6:**
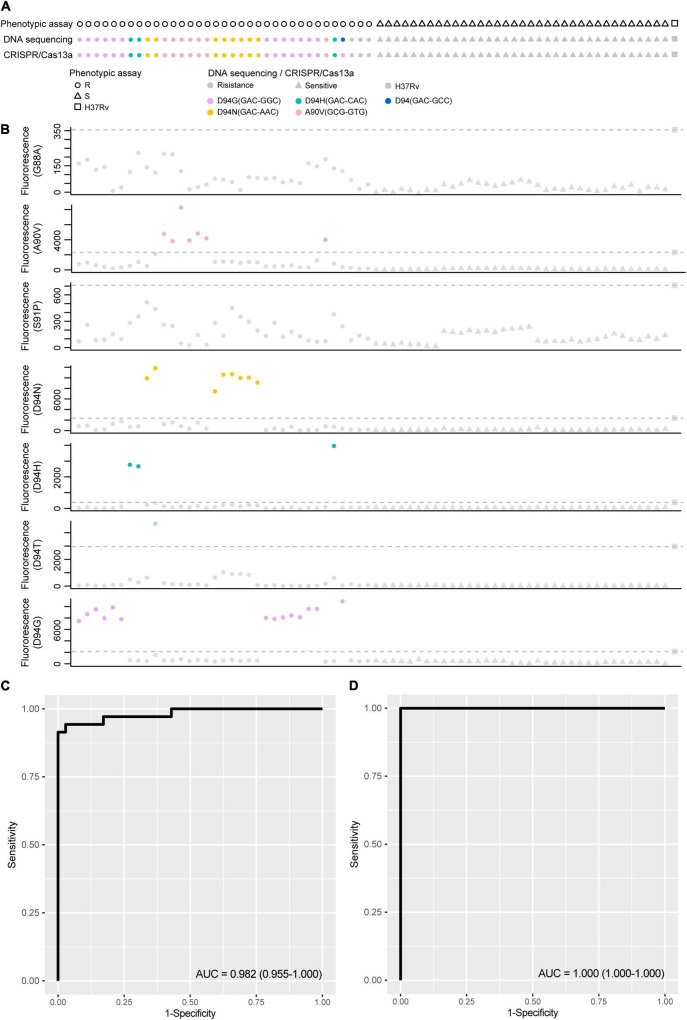
The performance of CRISPR-Cas13a systems for detection fluoroquinolone resistance related mutations compared with phenotypic resistance and DNA sequencing. (A) Fluoroquinolone resistance test results obtained from phenotypic based, DNA sequencing and CRISPR-Cas13a system in 75 clinical isolated *M. tuberculosis* strains. circular represent resistance result and triangle represent sensitive. Different color filled in circular represent strains with certain fluoroquinolone resistance. (B) The scatter plot of fluorescence value guided by different crRNA target to different mutations, dot lines represent positive threshold of each crRNA, and positive results were labeled with different color. **(C)** Diagnostic potential of CRISPR-Cas13a systems for detection fluoroquinolone resistance related mutations was evaluated by ROC (Receiver Operating Characteristic) curve analysis with AUC (Area under curve) within 95% confidence interval (CI), when phenotypic resistance was used as reference method. **(D)** Diagnostic potential of CRISPR-Cas13a systems for detection fluoroquinolone resistance related mutations was evaluated by ROC curve analysis with AUC within 95% confidence interval (CI), when DNA sequencing was used as reference method.

The application ability of LwCas13a was investigated by detecting genomic DNA from different clinical MTB strains. Genomic DNA of *M. tuberculosis* H37Rv was selected as the control sample. Seventy-five *M. tuberculosis* strains, including 35 fluoroquinolone-resistant (resistant to at least ofloxacin or levofloxacin) and 40 fluoroquinolone-sensitive strains, were collected from the Urumqi Municipal Centre for Disease Control and Prevention (Supplementary Table 3). The mutations in *gyrA* and drug resistance information are shown in Figure 6. Genomic DNA was extracted and diluted to 5 ng/μL in a PCR amplification system, and the T7 RNA polymerase promoter sequence was appended ahead of the PCR primer. PCR products were further analyzed by fluoroquinolone resistance mutation detection integrated with the T7 RNA polymerase transcription system and LwCas13a trans-cleavage RNase activity fluorescence detection system. crRNAs designed for different fluoroquinolone resistance SNPs were added to LwCas13a systems. The limitation of the current PCR-Cas13a detection system to distinguish the mutations from wild-type temples varied from 1 × 10^0^ copies/μL (S91P) to 1 × 10^2^ copies/μL (D94G), depending on the performance of different crRNAs (Supplementary Figure 3). The cut-off value of fluorescence to judge whether the sample contained certain mutations was set to the mean value plus 5 times the standard deviation based on *M. tuberculosis* H37Rv. In Figure 6, the sensitivity and specificity of the LwCas13a-based assay for the detection of fluoroquinolone phenotypic resistance were 91.43 and 100.00%, respectively. The positive prediction value in fluoroquinolone resistance mutation detection was 91.43% (95%CI, 76.94–98.20%) while the negative prediction value was 93.02% (95%CI, 80.94–98.54%) (Table 2). Three isolates that were found to be resistant by phenotypic drug susceptibility testing but found to be susceptible by the investigational assay were also found to be wild-type based on method of DNA sequencing. The sensitivity and specificity of the LwCas13a-based assay for detecting resistance mutations from DNA sequencing were 100 and 100%, respectively, in this small clinical sample cohort.

**TABLE 2:** The performance CRISPR-Cas13a systems in *M. tuberculosis* fluoroquinolone resistance mutation identification.

CRISPR-Cas13a system testing	Phenotypic drug-susceptibility testing		Total
	Positive (+)	Negative (−)	
Positive (+)	32	0	32
Negative (−)	3	40	43
Total	35	40	75
Performance with 95% CI	Sensitivity	Specificity	Consistency
	91.43% (76.94–98.20)	100.00% (91.19–100.00)	96.00% (88.75–99.17)
	PPV	NPV	
	100.00% (89.11–100.00)	93.02% (80.94–98.54)	

*PPV, positive prediction value; NPV, negative prediction value.*

## Discussion

CRISPR/Cas technology has been reported to show high off-target effects, which impedes its applications in mutation recognition, and there is a lack of rational guidelines for high-specificity Cas13a-crRNA design. In this study, we developed a crRNA screening strategy to increase the specificity of the CRISPR/Cas13a system, providing high resolution (8.29–38.22) for dramatically distinguishing fluoroquinolone-resistant mutations from their wild-type homologs.

Introducing a mismatch in the 5′ region of the crRNA spacer could form a “bubble” or distortion of the crRNA/target RNA hybrid and hinder the HEPN-nuclease activity of Cas13a, this providing the Cas13a platform with single nucleotide resolution when distinguishing the target RNA from its homologues (Gootenberg et al., 2017; Zhou et al., 2020). Introducing an extra secondary structure in the crRNA (hairpin-spacer crRNAs) suppressed the off-target nuclease activity of Cas13a to decrease the binding affinity to off-target ssRNA and increased the discrimination factor by twofold compared with ordinary crRNA (Ke et al., 2021). In this study, based on the rational introduction of a synthetic mismatch in the 5′ region of a crRNA, we obtained crRNAs that robustly distinguished SNPs that led to aa substitutions in GyrA G88A and A90V, with specificity ratios ranging from 38.22 to 8.29. For certain target SNPs, rationally designed crRNAs exhibited weak selectivity for single-base distinction over perfectly matched crRNAs (SNPs led aa substitutions in GyrA S91P and D94N, H, T, and G), and the target mismatch-anchoring crRNA screening strategy was proposed to obtain the optimal collocation between the target mismatch and synthetic mismatch to yield the robust discrimination ability of crRNAs used for SNP recognition. Moreover, thermodynamic titrations of the binding affinity for different crRNAs with target mismatches and synthetic mismatches in different positions toward the Cas13a protein may provide more clues (Ke et al., 2021).

Here, the clinical performance of our Cas13a-based molecular fluoroquinolone susceptibility tests resulted in 91.4% sensitivity and 100% specificity when the phenotypic drug-susceptibility testing was considered as the reference comparator, and 100% sensitivity and 100% specificity when genetic mutation was the reference standard, considering this small clinical evaluation based on purified cultured DNA as the input sample. There were 3 strains were found to be resistant by phenotypic drug-susceptibility testing but were found to be wild-type by current assay were also found to be wild-type by DNA sequencing, this result is similar with previously studies in which also other assays were used for *M. tuberculosis* drug-susceptibility testing (Brossier et al., 2010; Molina-Moya et al., 2015; Tagliani et al., 2015). The phenotypic-genotypic discrepancies at least caused by alternative molecular mechanisms of resistance and the limitations of the critical-concentration methods used for phenotypic testing.

Our study has several limitations. First, the investigational assay developed here is an assay based on purified PCR product, the integration system, including quick DNA extraction, recombinase polymerase amplification (RPA) and portable deployment for readout (Zhou et al., 2020; Sam et al., 2021), will make this platform easier to detect the mutations in a range of applications. Second, the clinical performance of current investigational assay still need sputum based evaluation, and whether the performance met the target for diagnostic sensitivity and specificity of next-generation molecular drug susceptibility tests provided by the WHO still need more evaluation (World Health Organization [Who], 2014), the clinical performance also need be done more to compare with current commercial assay, such as BACTEC™ MGIT™ 960 system and Cepheid Xpert^®^ MTB/XDR. Finally, the investigational assay was developed to detection classic fluoroquinolone resistance mutation, novel mutations led fluoroquinolone resistance phenotype would be miss out by current assay.

In summary, a crRNA screening strategy was proposed, and a set of crRNAs with robust resolving power in MTB fluoroquinolone resistance mutations were obtained. This CRISPR-Cas13a-based SNP detection system possessed several advantages, such as time savings and easy operation. Combined with optimized sample extraction and amplification, our system will facilitate further point-of-care molecular diagnostics for MTB drug susceptibility testing and facilitate more precise drug treatment in patients with TB.

## Data Availability Statement

The original contributions presented in the study are included in the article/Supplementary Material, further inquiries can be directed to the corresponding author/s.

## Author Contributions

QL, AH, YQL, and YLL conceived and designed the study. XB, PG, KQ, and JY conducted the experiment. HD, HZ, and MH analyzed the data. QL and XB wrote the manuscript. YH revised the manuscript. All authors contributed to the article and approved the submitted version.

## Conflict of Interest

The authors declare that the research was conducted in the absence of any commercial or financial relationships that could be construed as a potential conflict of interest.

## Publisher’s Note

All claims expressed in this article are solely those of the authors and do not necessarily represent those of their affiliated organizations, or those of the publisher, the editors and the reviewers. Any product that may be evaluated in this article, or claim that may be made by its manufacturer, is not guaranteed or endorsed by the publisher.

## Footnotes

1
http://www.addgene.org/90097/

## Supplementary Material

Supplementary Figure 1 Target mutation anchored screening strategy enables a robust resolving power of SNPs led GyrA S91P aa substitution. (A) Depiction of crRNA design strategy for the first-round screening. (B) Collateral cleavage activity of crRNAs from first-round screening on wt ssRNA or mutant RNA at different positions. (C) Specificity ratios are calculated as the ratio of the mutant ssRNA (on target) collateral cleavage to the wildtype ssRNA (off target) collateral cleavage. crRNAs that contain target mutation were anchored in region B, while the synthetic mismatch introduced in regions A and C was higher than in other combinations. (D) Depiction of crRNAs generated from fine-tuning for position combinations of target mismatch and synthetic mismatch. (E) Collateral cleavage activity of crRNAs from fine-tuning process on wt ssRNA or mutant RNA. (F) Specificity ratios of crRNAs after fine-tuning on wt ssRNA and on mutant ssRNA.

Supplementary Figure 2 Target mutation anchored screening strategy enables a robust resolving power of SNPs led GyrA D94G aa substitution. (A) Depiction of crRNA design strategy for the first-round screening. (B) Collateral cleavage activity of crRNAs from first-round screening on wt ssRNA or mutant RNA at different positions. (C) Specificity ratios are calculated as the ratio of the mutant ssRNA (on target) collateral cleavage to the wildtype ssRNA (off target) collateral cleavage. crRNAs that contain target mutation were anchored in region C, while the synthetic mismatch introduced in region B was higher than in other combinations. (D) Depiction of crRNAs generated from fine-tuning for position combinations of target mismatch and synthetic mismatch. (E) Collateral cleavage activity of crRNAs from fine-tuning process on wt ssRNA or mutant RNA. (F) Specificity ratios of crRNAs after fine-tuning on wt ssRNA and on mutant ssRNA.

Supplementary Figure 3 The sensitivity of PCR-LwCas13a systems in detection of fluoroquinolone resistance.
