# Supplementary figures and images for: A Highly Sensitive and Specific Detection Method for Mycobacterium tuberculosis Fluoroquinolone Resistance Mutations Utilizing the CRISPR-Cas13a System
Source: Front Microbiol. 2022 May 13;13:847373. doi: 10.3389/fmicb.2022.847373 (PMC9136396; doi:10.3389/fmicb.2022.847373)

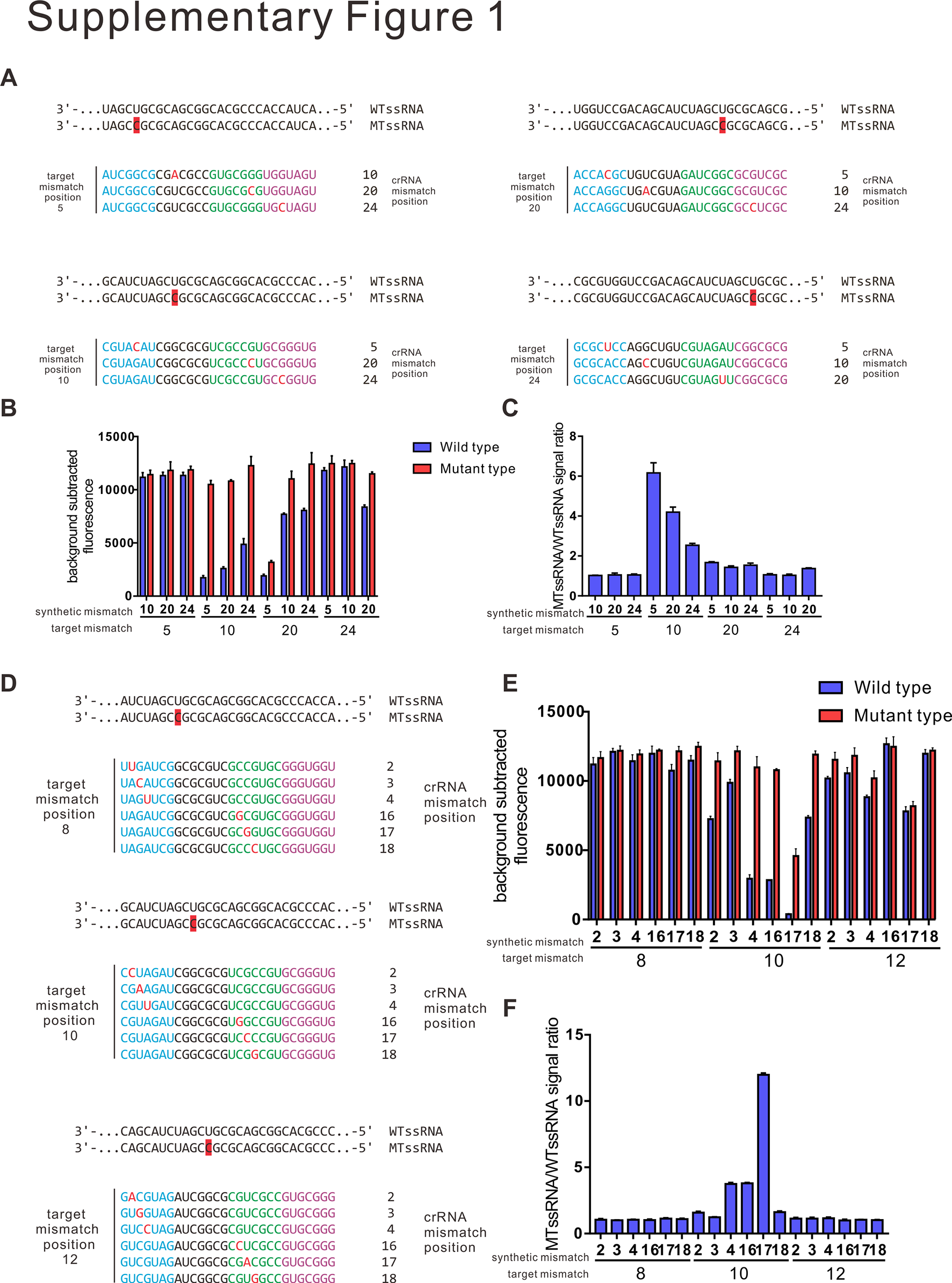

Supplement: Supplementary Figure 1 — Target mutation anchored screening strategy enables a robust resolving power of SNPs led GyrA S91P aa substitution. (A) Depiction of crRNA design strategy for the first-round screening. (B) Collateral cleavage activity of crRNAs from first-round screening on wt ssRNA or mutant RNA at different positions. (C) Specificity ratios are calculated as the ratio of the mutant ssRNA (on target) collateral cleavage to the wildtype ssRNA (off target) collateral cleavage. crRNAs that contain target mutation were anchored in region B, while the synthetic mismatch introduced in regions A and C was higher than in other combinations. (D) Depiction of crRNAs generated from fine-tuning for position combinations of target mismatch and synthetic mismatch. (E) Collateral cleavage activity of crRNAs from fine-tuning process on wt ssRNA or mutant RNA. (F) Specificity ratios of crRNAs after fine-tuning on wt ssRNA and on mutant ssRNA. [file Image_1.TIF]

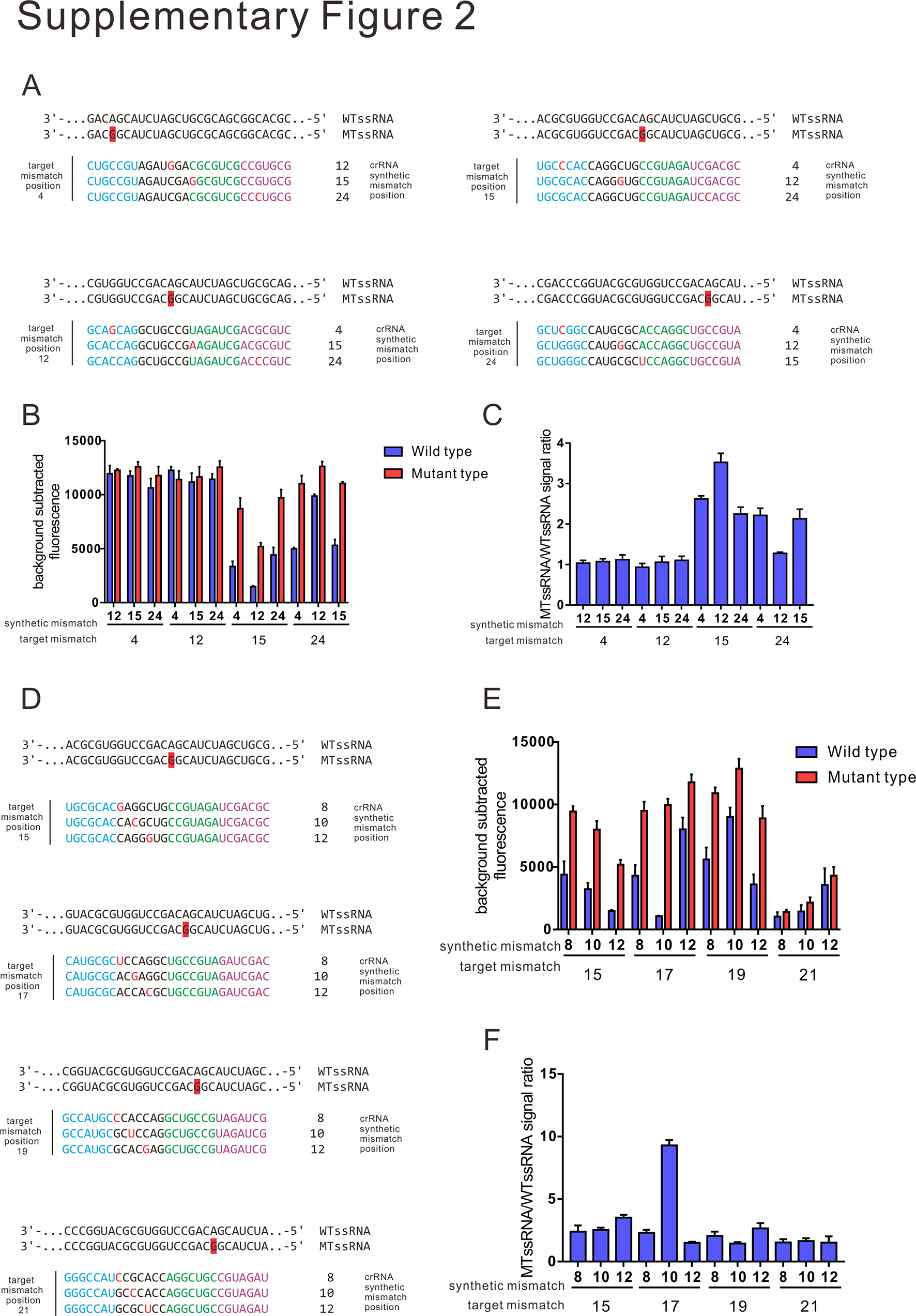

Supplement: Supplementary Figure 2 — Target mutation anchored screening strategy enables a robust resolving power of SNPs led GyrA D94G aa substitution. (A) Depiction of crRNA design strategy for the first-round screening. (B) Collateral cleavage activity of crRNAs from first-round screening on wt ssRNA or mutant RNA at different positions. (C) Specificity ratios are calculated as the ratio of the mutant ssRNA (on target) collateral cleavage to the wildtype ssRNA (off target) collateral cleavage. crRNAs that contain target mutation were anchored in region C, while the synthetic mismatch introduced in region B was higher than in other combinations. (D) Depiction of crRNAs generated from fine-tuning for position combinations of target mismatch and synthetic mismatch. (E) Collateral cleavage activity of crRNAs from fine-tuning process on wt ssRNA or mutant RNA. (F) Specificity ratios of crRNAs after fine-tuning on wt ssRNA and on mutant ssRNA. [file Image_2.TIF]

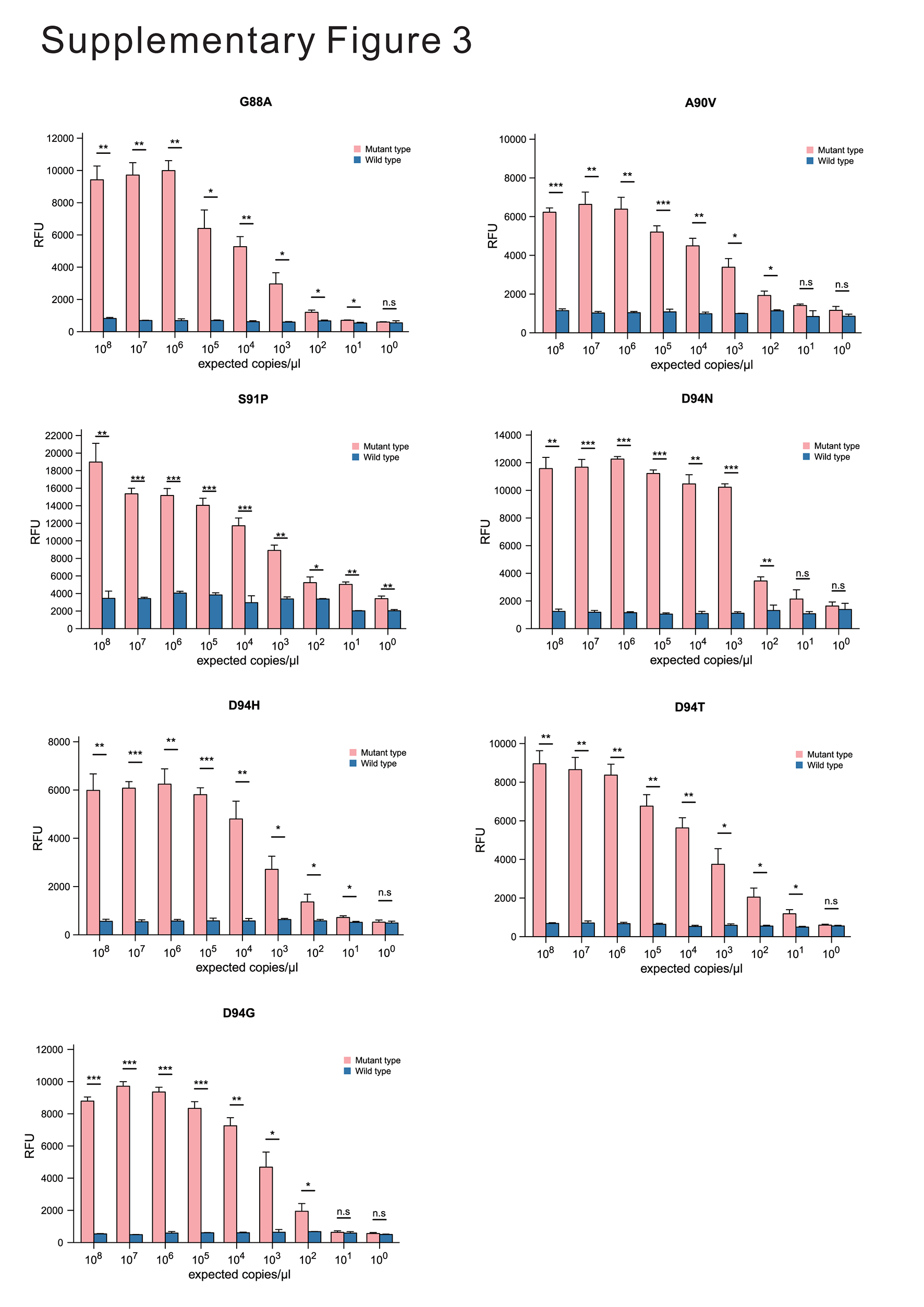

Supplement: Supplementary Figure 3 — The sensitivity of PCR-LwCas13a systems in detection of fluoroquinolone resistance. [file Image_3.TIF]
