# Supplementary material for: A Highly Sensitive and Specific Detection Method for Mycobacterium tuberculosis Fluoroquinolone Resistance Mutations Utilizing the CRISPR-Cas13a System
Source: Front Microbiol. 2022 May 13;13:847373. doi: 10.3389/fmicb.2022.847373 (PMC9136396; doi:10.3389/fmicb.2022.847373)
Supplement: Supplementary file 4 [file Table_1.docx]

**Supplementary Table 1. QRDR sequence and primers used for sit mutation introduction in QRDR.**

| Name | Sequence (5' to 3') |
| --- | --- |
| QRDR Sequence | GTGCGCGACGGGCTCAAGCCCGTGCATCGCCGGGTGCTCTATGCAATGTTCGATTCCGGCTTCCGCCCGGACCGCAGCCACGCCAAGTCGGCCCGGTCGGTTGCCGAGACCATGGGCAACTACCACCCGCACGGCGACGCGTCGATCTACGACAGCCTGGTGCGCATGGCCCAGCCCTGGTCGCTGCGCTACCCGCTGGTGGACGGCCAGGGCAACTTCGGCTCGCCAGGCAATGACCCACCGGCGGCGATGAGGTACACCGAAGCCCGGCTGACCCCGTTGGCGATGGAGATGCTGAGG |
| gyrA G88A F | AACTACCACCCGCACGCCGACGCGTCGAT |
| gyrA G88A R | GCGTGCGGGTGGTAGTTGCCCATGGTCT |
| gyrA A90V F | ACCCGCACGGCGACGTGTCGATCTACGACA |
| gyrA A90V R | ACGTCGCCGTGCGGGTGGTAGTTGCCCAT |
| gyrA S91P F | ACCCGCACGGCGACGCGCCGATCTACGACA |
| gyrA S91P R | GCGCGTCGCCGTGCGGGTGGTAGTTGCCCAT |
| gyrA D94N F | GGCGACGCGTCGATCTACAACAGCCTGGTGC |
| gyrA D94N R | TGTAGATCGACGCGTCGCCGTGCGGGTGGTA |
| gyrA D94H F | GACGCGTCGATCTACCACAGCCTGGTGCG |
| gyrA D94H R | GGTAGATCGACGCGTCGCCGTGCGGGT |
| gyrA D94Y F | GACGCGTCGATCTACTACAGCCTGGTGC |
| gyrA D94Y R | AGTAGATCGACGCGTCGCCGTGCGGGT |
| gyrA D94G F | GACGCGTCGATCTACGGCAGCCTGGTGC |
| gyrA D94G R | CCGTAGATCGACGCGTCGCCGTGCGGGT |
| T7 RNA polymerase promoter containing QRDR F | AATCTAATACGACTCACTATAGGGTGCTCTATGCAATGTTC |
| QRDR R | GAAGTTGCCCTGGCCGTCCACC |

QRDR, quinolones resistance determine region. PCR product amplified with T7 RNA polymerase promoter containing QRDR F and QRDR R were further used for target ssRNA preparation via T7 RNA polymerase transcription.
