# Supplementary material for: A Highly Sensitive and Specific Detection Method for Mycobacterium tuberculosis Fluoroquinolone Resistance Mutations Utilizing the CRISPR-Cas13a System
Source: Front Microbiol. 2022 May 13;13:847373. doi: 10.3389/fmicb.2022.847373 (PMC9136396; doi:10.3389/fmicb.2022.847373)
Supplement: Supplementary file 5 [file Table_2.docx]

**Supplementary Table 2. ssDNA templates for crRNA production**

| Name | Sequence (5' to 3') |
| --- | --- |
| G88A 3-1crRNA ssDNA template | ACCATGGGCAACTACCACCCGCACG**C**C**C**GTTTTAGTCCCCTTCGTTTTTGGGGTAGTCTAAATCCCCTATAGTGAGTCGTATTAGCTT |
| G88A 3-2crRNA ssDNA template | ACCATGGGCAACTACCACCCGCACG**CG**GGTTTTAGTCCCCTTCGTTTTTGGGGTAGTCTAAATCCCCTATAGTGAGTCGTATTAGCTT |
| G88A 3-4crRNA ssDNA template | ACCATGGGCAACTACCACCCGCAC**CC**CGGTTTTAGTCCCCTTCGTTTTTGGGGTAGTCTAAATCCCCTATAGTGAGTCGTATTAGCTT |
| G88A 3-5crRNA ssDNA template | ACCATGGGCAACTACCACCCGCA**G**G**C**CGGTTTTAGTCCCCTTCGTTTTTGGGGTAGTCTAAATCCCCTATAGTGAGTCGTATTAGCTT |
| G88A 3-6crRNA ssDNA template | ACCATGGGCAACTACCACCCGC**T**CG**C**CGGTTTTAGTCCCCTTCGTTTTTGGGGTAGTCTAAATCCCCTATAGTGAGTCGTATTAGCTT |
| G88A 4-1crRNA ssDNA template | CCATGGGCAACTACCACCCGCACG**C**CG**T**GTTTTAGTCCCCTTCGTTTTTGGGGTAGTCTAAATCCCCTATAGTGAGTCGTATTAGCTT |
| G88A 4-2crRNA ssDNA template | CCATGGGCAACTACCACCCGCACG**C**C**C**AGTTTTAGTCCCCTTCGTTTTTGGGGTAGTCTAAATCCCCTATAGTGAGTCGTATTAGCTT |
| G88A 4-3crRNA ssDNA template | CCATGGGCAACTACCACCCGCACG**CG**GAGTTTTAGTCCCCTTCGTTTTTGGGGTAGTCTAAATCCCCTATAGTGAGTCGTATTAGCTT |
| G88A 4-5crRNA ssDNA template | CCATGGGCAACTACCACCCGCAC**CC**CGAGTTTTAGTCCCCTTCGTTTTTGGGGTAGTCTAAATCCCCTATAGTGAGTCGTATTAGCTT |
| G88A 4-6crRNA ssDNA template | CCATGGGCAACTACCACCCGCA**G**G**C**CGAGTTTTAGTCCCCTTCGTTTTTGGGGTAGTCTAAATCCCCTATAGTGAGTCGTATTAGCTT |
| G88A 4-7crRNA ssDNA template | CCATGGGCAACTACCACCCGC**T**CG**C**CGAGTTTTAGTCCCCTTCGTTTTTGGGGTAGTCTAAATCCCCTATAGTGAGTCGTATTAGCTT |
| G88A 5-2crRNA ssDNA template | CATGGGCAACTACCACCCGCACG**C**CG**T**CGTTTTAGTCCCCTTCGTTTTTGGGGTAGTCTAAATCCCCTATAGTGAGTCGTATTAGCTT |
| G88A 5-3crRNA ssDNA template | CATGGGCAACTACCACCCGCACG**C**C**C**ACGTTTTAGTCCCCTTCGTTTTTGGGGTAGTCTAAATCCCCTATAGTGAGTCGTATTAGCTT |
| G88A 5-4crRNA ssDNA template | CATGGGCAACTACCACCCGCACG**CG**GACGTTTTAGTCCCCTTCGTTTTTGGGGTAGTCTAAATCCCCTATAGTGAGTCGTATTAGCTT |
| G88A 5-6crRNA ssDNA template | CATGGGCAACTACCACCCGCAC**CC**CGACGTTTTAGTCCCCTTCGTTTTTGGGGTAGTCTAAATCCCCTATAGTGAGTCGTATTAGCTT |
| G88A 5-7crRNA ssDNA template | CATGGGCAACTACCACCCGCA**G**G**C**CGACGTTTTAGTCCCCTTCGTTTTTGGGGTAGTCTAAATCCCCTATAGTGAGTCGTATTAGCTT |
| G88A 5-8crRNA ssDNA template | CATGGGCAACTACCACCCGC**T**CG**C**CGACGTTTTAGTCCCCTTCGTTTTTGGGGTAGTCTAAATCCCCTATAGTGAGTCGTATTAGCTT |
| G88A 6-3crRNA ssDNA template | ATGGGCAACTACCACCCGCACG**C**CG**T**CGGTTTTAGTCCCCTTCGTTTTTGGGGTAGTCTAAATCCCCTATAGTGAGTCGTATTAGCTT |
| G88A 6-4crRNA ssDNA template | ATGGGCAACTACCACCCGCACG**C**C**C**ACGGTTTTAGTCCCCTTCGTTTTTGGGGTAGTCTAAATCCCCTATAGTGAGTCGTATTAGCTT |
| G88A 6-5crRNA ssDNA template | ATGGGCAACTACCACCCGCACG**CG**GACGGTTTTAGTCCCCTTCGTTTTTGGGGTAGTCTAAATCCCCTATAGTGAGTCGTATTAGCTT |
| G88A 6-7crRNA ssDNA template | ATGGGCAACTACCACCCGCAC**CC**CGACGGTTTTAGTCCCCTTCGTTTTTGGGGTAGTCTAAATCCCCTATAGTGAGTCGTATTAGCTT |
| G88A 6-8crRNA ssDNA template | ATGGGCAACTACCACCCGCA**G**G**C**CGACGGTTTTAGTCCCCTTCGTTTTTGGGGTAGTCTAAATCCCCTATAGTGAGTCGTATTAGCTT |
| G88A 6-9crRNA ssDNA template | ATGGGCAACTACCACCCGC**T**CG**C**CGACGGTTTTAGTCCCCTTCGTTTTTGGGGTAGTCTAAATCCCCTATAGTGAGTCGTATTAGCTT |
| A90V 4-6crRNA ssDNA template | GCAACTACCACCCGCACGGCGA**G**G**T**GTCGTTTTAGTCCCCTTCGTTTTTGGGGTAGTCTAAATCCCCTATAGTGAGTCGTATTAGCTT |
| S91P 4-6crRNA ssDNA template | AACTACCACCCGCACGGCGACG**G**G**C**CGAGTTTTAGTCCCCTTCGTTTTTGGGGTAGTCTAAATCCCCTATAGTGAGTCGTATTAGCTT |
| D94N 4-6crRNA ssDNA template | CCGCACGGCGACGCGTCGATCT**T**C**A**ACAGTTTTAGTCCCCTTCGTTTTTGGGGTAGTCTAAATCCCCTATAGTGAGTCGTATTAGCTT |
| D94H 4-6crRNA ssDNA template | CCGCACGGCGACGCGTCGATCT**T**C**C**ACAGTTTTAGTCCCCTTCGTTTTTGGGGTAGTCTAAATCCCCTATAGTGAGTCGTATTAGCTT |
| D94T 4-6crRNA ssDNA template | CCGCACGGCGACGCGTCGATCT**T**C**T**ACAGTTTTAGTCCCCTTCGTTTTTGGGGTAGTCTAAATCCCCTATAGTGAGTCGTATTAGCTT |
| D94G 4-6crRNA ssDNA template | CGCACGGCGACGCGTCGATCTA**G**G**G**CAGGTTTTAGTCCCCTTCGTTTTTGGGGTAGTCTAAATCCCCTATAGTGAGTCGTATTAGCTT |
| A90V 3-5crRNA ssDNA template | GGCAACTACCACCCGCACGGCGA**G**G**T**GTGTTTTAGTCCCCTTCGTTTTTGGGGTAGTCTAAATCCCCTATAGTGAGTCGTATTAGCTT |
| S91P 3-5crRNA ssDNA template | CAACTACCACCCGCACGGCGACG**G**G**C**CGGTTTTAGTCCCCTTCGTTTTTGGGGTAGTCTAAATCCCCTATAGTGAGTCGTATTAGCTT |
| D94N 3-5crRNA ssDNA template | CCCGCACGGCGACGCGTCGATCT**T**C**A**ACGTTTTAGTCCCCTTCGTTTTTGGGGTAGTCTAAATCCCCTATAGTGAGTCGTATTAGCTT |
| D94H 3-5crRNA ssDNA template | CCCGCACGGCGACGCGTCGATCT**T**C**C**ACGTTTTAGTCCCCTTCGTTTTTGGGGTAGTCTAAATCCCCTATAGTGAGTCGTATTAGCTT |
| D94T 3-5crRNA ssDNA template | CCCGCACGGCGACGCGTCGATCT**T**C**T**ACGTTTTAGTCCCCTTCGTTTTTGGGGTAGTCTAAATCCCCTATAGTGAGTCGTATTAGCTT |
| D94G 3-5crRNA ssDNA template | CCGCACGGCGACGCGTCGATCTA**G**G**G**CAGTTTTAGTCCCCTTCGTTTTTGGGGTAGTCTAAATCCCCTATAGTGAGTCGTATTAGCTT |
| D94N 4-10crRNA ssDNA template | CCGCACGGCGACGCGTCG**T**TCTAC**A**ACAGTTTTAGTCCCCTTCGTTTTTGGGGTAGTCTAAATCCCCTATAGTGAGTCGTATTAGCTT |
| D94N 4-17crRNA ssDNA template | CCGCACGGCGA**G**GCGTCGATCTAC**A**ACAGTTTTAGTCCCCTTCGTTTTTGGGGTAGTCTAAATCCCCTATAGTGAGTCGTATTAGCTT |
| D94N 4-24crRNA ssDNA template | CCGC**T**CGGCGACGCGTCGATCTAC**A**ACAGTTTTAGTCCCCTTCGTTTTTGGGGTAGTCTAAATCCCCTATAGTGAGTCGTATTAGCTT |
| D94N 10-4crRNA ssDNA template | GGCGACGCGTCGATCTAC**A**ACAGC**G**TGGGTTTTAGTCCCCTTCGTTTTTGGGGTAGTCTAAATCCCCTATAGTGAGTCGTATTAGCTT |
| D94N 10-17crRNA ssDNA template | GGCGACGCGTC**C**ATCTAC**A**ACAGCCTGGGTTTTAGTCCCCTTCGTTTTTGGGGTAGTCTAAATCCCCTATAGTGAGTCGTATTAGCTT |
| D94N 10-24crRNA ssDNA template | GGCG**T**CGCGTCGATCTAC**A**ACAGCCTGGGTTTTAGTCCCCTTCGTTTTTGGGGTAGTCTAAATCCCCTATAGTGAGTCGTATTAGCTT |
| D94N 17-4crRNA ssDNA template | CGTCGATCTAC**A**ACAGCCTGGTGC**C**CATGTTTTAGTCCCCTTCGTTTTTGGGGTAGTCTAAATCCCCTATAGTGAGTCGTATTAGCTT |
| D94N 17-10crRNA ssDNA template | CGTCGATCTAC**A**ACAGCC**A**GGTGCGCATGTTTTAGTCCCCTTCGTTTTTGGGGTAGTCTAAATCCCCTATAGTGAGTCGTATTAGCTT |
| D94N 17-24crRNA ssDNA template | CGTC**C**ATCTAC**A**ACAGCCTGGTGCGCATGTTTTAGTCCCCTTCGTTTTTGGGGTAGTCTAAATCCCCTATAGTGAGTCGTATTAGCTT |
| D94N 24-4crRNA ssDNA template | CTAC**A**ACAGCCTGGTGCGCATGGC**G**CAGGTTTTAGTCCCCTTCGTTTTTGGGGTAGTCTAAATCCCCTATAGTGAGTCGTATTAGCTT |
| D94N 24-10crRNA ssDNA template | CTAC**A**ACAGCCTGGTGCG**G**ATGGCCCAGGTTTTAGTCCCCTTCGTTTTTGGGGTAGTCTAAATCCCCTATAGTGAGTCGTATTAGCTT |
| D94N 24-17crRNA ssDNA template | CTAC**A**ACAGCC**A**GGTGCGCATGGCCCAGGTTTTAGTCCCCTTCGTTTTTGGGGTAGTCTAAATCCCCTATAGTGAGTCGTATTAGCTT |
| D94N 8-2crRNA ssDNA template | ACGGCGACGCGTCGATCTAC**A**ACAGC**G**TGTTTTAGTCCCCTTCGTTTTTGGGGTAGTCTAAATCCCCTATAGTGAGTCGTATTAGCTT |
| D94N 8-4crRNA ssDNA template | ACGGCGACGCGTCGATCTAC**A**ACA**C**CCTGTTTTAGTCCCCTTCGTTTTTGGGGTAGTCTAAATCCCCTATAGTGAGTCGTATTAGCTT |
| D94N 8-6crRNA ssDNA template | ACGGCGACGCGTCGATCTAC**A**A**G**AGCCTGTTTTAGTCCCCTTCGTTTTTGGGGTAGTCTAAATCCCCTATAGTGAGTCGTATTAGCTT |
| D94N 8-16crRNA ssDNA template | ACGGCGACGCGT**G**GATCTAC**A**ACAGCCTGTTTTAGTCCCCTTCGTTTTTGGGGTAGTCTAAATCCCCTATAGTGAGTCGTATTAGCTT |
| D94N 8-18crRNA ssDNA template | ACGGCGACGC**C**TCGATCTAC**A**ACAGCCTGTTTTAGTCCCCTTCGTTTTTGGGGTAGTCTAAATCCCCTATAGTGAGTCGTATTAGCTT |
| D94N 8-20crRNA ssDNA template | ACGGCGAC**C**CGTCGATCTAC**A**ACAGCCTGTTTTAGTCCCCTTCGTTTTTGGGGTAGTCTAAATCCCCTATAGTGAGTCGTATTAGCTT |
| D94N 10-2crRNA ssDNA template | GGCGACGCGTCGATCTAC**A**ACAGCCT**C**GGTTTTAGTCCCCTTCGTTTTTGGGGTAGTCTAAATCCCCTATAGTGAGTCGTATTAGCTT |
| D94N 10-6crRNA ssDNA template | GGCGACGCGTCGATCTAC**A**ACA**C**CCTGGGTTTTAGTCCCCTTCGTTTTTGGGGTAGTCTAAATCCCCTATAGTGAGTCGTATTAGCTT |
| D94N 10-18crRNA ssDNA template | GGCGACGCGT**G**GATCTAC**A**ACAGCCTGGGTTTTAGTCCCCTTCGTTTTTGGGGTAGTCTAAATCCCCTATAGTGAGTCGTATTAGCTT |
| D94N 10-20crRNA ssDNA template | GGCGACGC**C**TCGATCTAC**A**ACAGCCTGGGTTTTAGTCCCCTTCGTTTTTGGGGTAGTCTAAATCCCCTATAGTGAGTCGTATTAGCTT |
| D94N 12-2crRNA ssDNA template | CGACGCGTCGATCTAC**A**ACAGCCTGG**A**GGTTTTAGTCCCCTTCGTTTTTGGGGTAGTCTAAATCCCCTATAGTGAGTCGTATTAGCTT |
| D94N 12-4crRNA ssDNA template | CGACGCGTCGATCTAC**A**ACAGCCT**C**GTGGTTTTAGTCCCCTTCGTTTTTGGGGTAGTCTAAATCCCCTATAGTGAGTCGTATTAGCTT |
| D94N 12-6crRNA ssDNA template | CGACGCGTCGATCTAC**A**ACAGC**G**TGGTGGTTTTAGTCCCCTTCGTTTTTGGGGTAGTCTAAATCCCCTATAGTGAGTCGTATTAGCTT |
| D94N 12-16crRNA ssDNA template | CGACGCGTCGAT**G**TAC**A**ACAGCCTGGTGGTTTTAGTCCCCTTCGTTTTTGGGGTAGTCTAAATCCCCTATAGTGAGTCGTATTAGCTT |
| D94N 12-18crRNA ssDNA template | CGACGCGTCG**T**TCTAC**A**ACAGCCTGGTGGTTTTAGTCCCCTTCGTTTTTGGGGTAGTCTAAATCCCCTATAGTGAGTCGTATTAGCTT |
| D94N 12-20crRNA ssDNA template | CGACGCGT**G**GATCTAC**A**ACAGCCTGGTGGTTTTAGTCCCCTTCGTTTTTGGGGTAGTCTAAATCCCCTATAGTGAGTCGTATTAGCTT |
| S91P 10-16crRNA ssDNA template | CACCCGCACGGC**C**ACGCG**C**CGATCTACGGTTTTAGTCCCCTTCGTTTTTGGGGTAGTCTAAATCCCCTATAGTGAGTCGTATTAGCTT |
| D94H 10-16 crRNA ssDNA template | GGCGACGCGTCG**T**TCTAC**C**ACAGCCTGGGTTTTAGTCCCCTTCGTTTTTGGGGTAGTCTAAATCCCCTATAGTGAGTCGTATTAGCTT |
| D94T 10-16 crRNA ssDNA template | GGCGACGCGTCG**T**TCTAC**T**ACAGCCTGGGTTTTAGTCCCCTTCGTTTTTGGGGTAGTCTAAATCCCCTATAGTGAGTCGTATTAGCTT |
| D94G 10-16crRNA ssDNA template | GCGACGCGTCGA**A**CTACG**G**CAGCCTGGTGTTTTAGTCCCCTTCGTTTTTGGGGTAGTCTAAATCCCCTATAGTGAGTCGTATTAGCTT |
| S91P 5-10crRNA ssDNA template | ACTACCACCCGCACGGCG**T**CGCG**C**CGATGTTTTAGTCCCCTTCGTTTTTGGGGTAGTCTAAATCCCCTATAGTGAGTCGTATTAGCTT |
| S91P 5-20crRNA ssDNA template | ACTACCAC**G**CGCACGGCGACGCG**C**CGATGTTTTAGTCCCCTTCGTTTTTGGGGTAGTCTAAATCCCCTATAGTGAGTCGTATTAGCTT |
| S91P 5-24crRNA ssDNA template | ACTA**G**CACCCGCACGGCGACGCG**C**CGATGTTTTAGTCCCCTTCGTTTTTGGGGTAGTCTAAATCCCCTATAGTGAGTCGTATTAGCTT |
| S91P 10-5crRNA ssDNA template | CACCCGCACGGCGACGCG**C**CGAT**G**TACGGTTTTAGTCCCCTTCGTTTTTGGGGTAGTCTAAATCCCCTATAGTGAGTCGTATTAGCTT |
| S91P 10-20crRNA ssDNA template | CACCCGCA**G**GGCGACGCG**C**CGATCTACGGTTTTAGTCCCCTTCGTTTTTGGGGTAGTCTAAATCCCCTATAGTGAGTCGTATTAGCTT |
| S91P 10-24crRNA ssDNA template | CACC**G**GCACGGCGACGCG**C**CGATCTACGGTTTTAGTCCCCTTCGTTTTTGGGGTAGTCTAAATCCCCTATAGTGAGTCGTATTAGCTT |
| S91P 20-5crRNA ssDNA template | GCGACGCG**C**CGATCTACGACAGC**G**TGGTGTTTTAGTCCCCTTCGTTTTTGGGGTAGTCTAAATCCCCTATAGTGAGTCGTATTAGCTT |
| S91P 20-10crRNA ssDNA template | GCGACGCG**C**CGATCTACG**T**CAGCCTGGTGTTTTAGTCCCCTTCGTTTTTGGGGTAGTCTAAATCCCCTATAGTGAGTCGTATTAGCTT |
| S91P 20-24crRNA ssDNA template | GCGA**G**GCG**C**CGATCTACGACAGCCTGGTGTTTTAGTCCCCTTCGTTTTTGGGGTAGTCTAAATCCCCTATAGTGAGTCGTATTAGCTT |
| S91P 24-5crRNA ssDNA template | CGCG**C**CGATCTACGACAGCCTGG**A**GCGCGTTTTAGTCCCCTTCGTTTTTGGGGTAGTCTAAATCCCCTATAGTGAGTCGTATTAGCTT |
| S91P 24-10crRNA ssDNA template | CGCG**C**CGATCTACGACAG**G**CTGGTGCGCGTTTTAGTCCCCTTCGTTTTTGGGGTAGTCTAAATCCCCTATAGTGAGTCGTATTAGCTT |
| S91P 24-20crRNA ssDNA template | CGCG**C**CGA**A**CTACGACAGCCTGGTGCGCGTTTTAGTCCCCTTCGTTTTTGGGGTAGTCTAAATCCCCTATAGTGAGTCGTATTAGCTT |
| S91P 8-2crRNA ssDNA template | ACCACCCGCACGGCGACGCG**C**CGATC**A**AGTTTTAGTCCCCTTCGTTTTTGGGGTAGTCTAAATCCCCTATAGTGAGTCGTATTAGCTT |
| S91P 8-3crRNA ssDNA template | ACCACCCGCACGGCGACGCG**C**CGAT**G**TAGTTTTAGTCCCCTTCGTTTTTGGGGTAGTCTAAATCCCCTATAGTGAGTCGTATTAGCTT |
| S91P 8-4crRNA ssDNA template | ACCACCCGCACGGCGACGCG**C**CGA**A**CTAGTTTTAGTCCCCTTCGTTTTTGGGGTAGTCTAAATCCCCTATAGTGAGTCGTATTAGCTT |
| S91P 8-16crRNA ssDNA template | ACCACCCGCACG**C**CGACGCG**C**CGATCTAGTTTTAGTCCCCTTCGTTTTTGGGGTAGTCTAAATCCCCTATAGTGAGTCGTATTAGCTT |
| S91P 8-17crRNA ssDNA template | ACCACCCGCAC**C**GCGACGCG**C**CGATCTAGTTTTAGTCCCCTTCGTTTTTGGGGTAGTCTAAATCCCCTATAGTGAGTCGTATTAGCTT |
| S91P 8-18crRNA ssDNA template | ACCACCCGCA**G**GGCGACGCG**C**CGATCTAGTTTTAGTCCCCTTCGTTTTTGGGGTAGTCTAAATCCCCTATAGTGAGTCGTATTAGCTT |
| S91P 10-2crRNA ssDNA template | CACCCGCACGGCGACGCG**C**CGATCTA**G**GGTTTTAGTCCCCTTCGTTTTTGGGGTAGTCTAAATCCCCTATAGTGAGTCGTATTAGCTT |
| S91P 10-3crRNA ssDNA template | CACCCGCACGGCGACGCG**C**CGATCT**T**CGGTTTTAGTCCCCTTCGTTTTTGGGGTAGTCTAAATCCCCTATAGTGAGTCGTATTAGCTT |
| S91P 10-4crRNA ssDNA template | CACCCGCACGGCGACGCG**C**CGATC**A**ACGGTTTTAGTCCCCTTCGTTTTTGGGGTAGTCTAAATCCCCTATAGTGAGTCGTATTAGCTT |
| S91P 10-16crRNA ssDNA template | CACCCGCACGGC**C**ACGCG**C**CGATCTACGGTTTTAGTCCCCTTCGTTTTTGGGGTAGTCTAAATCCCCTATAGTGAGTCGTATTAGCTT |
| S91P 10-17crRNA ssDNA template | CACCCGCACGG**G**GACGCG**C**CGATCTACGGTTTTAGTCCCCTTCGTTTTTGGGGTAGTCTAAATCCCCTATAGTGAGTCGTATTAGCTT |
| S91P 10-18crRNA ssDNA template | CACCCGCACG**C**CGACGCG**C**CGATCTACGGTTTTAGTCCCCTTCGTTTTTGGGGTAGTCTAAATCCCCTATAGTGAGTCGTATTAGCTT |
| S91P 12-2crRNA ssDNA template | CCCGCACGGCGACGCG**C**CGATCTACG**T**CGTTTTAGTCCCCTTCGTTTTTGGGGTAGTCTAAATCCCCTATAGTGAGTCGTATTAGCTT |
| S91P 12-3crRNA ssDNA template | CCCGCACGGCGACGCG**C**CGATCTAC**C**ACGTTTTAGTCCCCTTCGTTTTTGGGGTAGTCTAAATCCCCTATAGTGAGTCGTATTAGCTT |
| S91P 12-4crRNA ssDNA template | CCCGCACGGCGACGCG**C**CGATCTA**G**GACGTTTTAGTCCCCTTCGTTTTTGGGGTAGTCTAAATCCCCTATAGTGAGTCGTATTAGCTT |
| S91P 12-16crRNA ssDNA template | CCCGCACGGCGA**G**GCG**C**CGATCTACGACGTTTTAGTCCCCTTCGTTTTTGGGGTAGTCTAAATCCCCTATAGTGAGTCGTATTAGCTT |
| S91P 12-17crRNA ssDNA template | CCCGCACGGCG**T**CGCG**C**CGATCTACGACGTTTTAGTCCCCTTCGTTTTTGGGGTAGTCTAAATCCCCTATAGTGAGTCGTATTAGCTT |
| S91P 12-18crRNA ssDNA template | CCCGCACGGC**C**ACGCG**C**CGATCTACGACGTTTTAGTCCCCTTCGTTTTTGGGGTAGTCTAAATCCCCTATAGTGAGTCGTATTAGCTT |
| D94G 4-12crRNA ssDNA template | CGCACGGCGACGCGTC**C**ATCTACG**G**CAGGTTTTAGTCCCCTTCGTTTTTGGGGTAGTCTAAATCCCCTATAGTGAGTCGTATTAGCTT |
| D94G 4-15crRNA ssDNA template | CGCACGGCGACGC**C**TCGATCTACG**G**CAGGTTTTAGTCCCCTTCGTTTTTGGGGTAGTCTAAATCCCCTATAGTGAGTCGTATTAGCTT |
| D94G 4-24crRNA ssDNA template | CGCA**G**GGCGACGCGTCGATCTACG**G**CAGGTTTTAGTCCCCTTCGTTTTTGGGGTAGTCTAAATCCCCTATAGTGAGTCGTATTAGCTT |
| D94G 12-4crRNA ssDNA template | GACGCGTCGATCTACG**G**CAGCCTG**C**TGCGTTTTAGTCCCCTTCGTTTTTGGGGTAGTCTAAATCCCCTATAGTGAGTCGTATTAGCTT |
| D94G 12-15crRNA ssDNA template | GACGCGTCGATCT**T**CG**G**CAGCCTGGTGCGTTTTAGTCCCCTTCGTTTTTGGGGTAGTCTAAATCCCCTATAGTGAGTCGTATTAGCTT |
| D94G 12-24crRNA ssDNA template | GACG**G**GTCGATCTACG**G**CAGCCTGGTGCGTTTTAGTCCCCTTCGTTTTTGGGGTAGTCTAAATCCCCTATAGTGAGTCGTATTAGCTT |
| D94G 15-4crRNA ssDNA template | GCGTCGATCTACG**G**CAGCCTGGTG**G**GCAGTTTTAGTCCCCTTCGTTTTTGGGGTAGTCTAAATCCCCTATAGTGAGTCGTATTAGCTT |
| D94G 15-12crRNA ssDNA template | GCGTCGATCTACG**G**CA**C**CCTGGTGCGCAGTTTTAGTCCCCTTCGTTTTTGGGGTAGTCTAAATCCCCTATAGTGAGTCGTATTAGCTT |
| D94G 15-24crRNA ssDNA template | GCGT**G**GATCTACG**G**CAGCCTGGTGCGCAGTTTTAGTCCCCTTCGTTTTTGGGGTAGTCTAAATCCCCTATAGTGAGTCGTATTAGCTT |
| D94G 24-4crRNA ssDNA template | TACG**G**CAGCCTGGTGCGCATGGCC**G**AGCGTTTTAGTCCCCTTCGTTTTTGGGGTAGTCTAAATCCCCTATAGTGAGTCGTATTAGCTT |
| D94G 24-12crRNA ssDNA template | TACG**G**CAGCCTGGTGC**C**CATGGCCCAGCGTTTTAGTCCCCTTCGTTTTTGGGGTAGTCTAAATCCCCTATAGTGAGTCGTATTAGCTT |
| D94G 24-15crRNA ssDNA template | TACG**G**CAGCCTGG**A**GCGCATGGCCCAGCGTTTTAGTCCCCTTCGTTTTTGGGGTAGTCTAAATCCCCTATAGTGAGTCGTATTAGCTT |
| D94G 15-8crRNA ssDNA template | GCGTCGATCTACG**G**CAGCCT**C**GTGCGCAGTTTTAGTCCCCTTCGTTTTTGGGGTAGTCTAAATCCCCTATAGTGAGTCGTATTAGCTT |
| D94G 15-10crRNA ssDNA template | GCGTCGATCTACG**G**CAGC**G**TGGTGCGCAGTTTTAGTCCCCTTCGTTTTTGGGGTAGTCTAAATCCCCTATAGTGAGTCGTATTAGCTT |
| D94G 17-8crRNA ssDNA template | GTCGATCTACG**G**CAGCCTGG**A**GCGCATGGTTTTAGTCCCCTTCGTTTTTGGGGTAGTCTAAATCCCCTATAGTGAGTCGTATTAGCTT |
| D94G 17-10crRNA ssDNA template | GTCGATCTACG**G**CAGCCT**C**GTGCGCATGGTTTTAGTCCCCTTCGTTTTTGGGGTAGTCTAAATCCCCTATAGTGAGTCGTATTAGCTT |
| D94G 17-12crRNA ssDNA template | GTCGATCTACG**G**CAGC**G**TGGTGCGCATGGTTTTAGTCCCCTTCGTTTTTGGGGTAGTCTAAATCCCCTATAGTGAGTCGTATTAGCTT |
| D94G 19-8crRNA ssDNA template | CGATCTACG**G**CAGCCTGGTG**G**GCATGGCGTTTTAGTCCCCTTCGTTTTTGGGGTAGTCTAAATCCCCTATAGTGAGTCGTATTAGCTT |
| D94G 19-10crRNA ssDNA template | CGATCTACG**G**CAGCCTGG**A**GCGCATGGCGTTTTAGTCCCCTTCGTTTTTGGGGTAGTCTAAATCCCCTATAGTGAGTCGTATTAGCTT |
| D94G 19-12crRNA ssDNA template | CGATCTACG**G**CAGCCT**C**GTGCGCATGGCGTTTTAGTCCCCTTCGTTTTTGGGGTAGTCTAAATCCCCTATAGTGAGTCGTATTAGCTT |
| D94G 21-8crRNA ssDNA template | ATCTACG**G**CAGCCTGGTGCG**G**ATGGCCCGTTTTAGTCCCCTTCGTTTTTGGGGTAGTCTAAATCCCCTATAGTGAGTCGTATTAGCTT |
| D94G 21-10crRNA ssDNA template | ATCTACG**G**CAGCCTGGTG**G**GCATGGCCCGTTTTAGTCCCCTTCGTTTTTGGGGTAGTCTAAATCCCCTATAGTGAGTCGTATTAGCTT |
| D94G 21-12crRNA ssDNA template | ATCTACG**G**CAGCCTGG**A**GCGCATGGCCCGTTTTAGTCCCCTTCGTTTTTGGGGTAGTCTAAATCCCCTATAGTGAGTCGTATTAGCTT |
| T7 RNA polymerase promoter anneal primer | AAGCTAATACGACTCACTATA |

Sequence of T7 RNA polymerase promoter was underlined. Target mutation and synthetic mismatches were in bold
