# Supplementary material for: A Highly Sensitive and Specific Detection Method for Mycobacterium tuberculosis Fluoroquinolone Resistance Mutations Utilizing the CRISPR-Cas13a System
Source: Front Microbiol. 2022 May 13;13:847373. doi: 10.3389/fmicb.2022.847373 (PMC9136396; doi:10.3389/fmicb.2022.847373)
Supplement: Supplementary file 6 [file Table_3.docx]

Supplemental Table 3. cross/mono resistance among drugs within the fluoroquinolone group

| Strains | fluoroquinolone resistance  (Phenotypic drug-susceptibility testing) | | |
| --- | --- | --- | --- |
| fluoroquinolones resistant strains | 4μg/ml OFX | 2μg/ml LFX | 1μg/ml MFX |
| 1 | R | R | R |
| 2 | R | R | R |
| 3 | R | R | S |
| 4 | R | R | R |
| 5 | R | R | R |
| 6 | R | R | R |
| 7 | R | R | R |
| 8 | R | R | S |
| 9 | R | R | R |
| 10 | R | R | R |
| 11 | R | R | R |
| 12 | R | R | R |
| 13 | R | R | R |
| 14 | R | R | S |
| 15 | R | R | R |
| 16 | R | R | R |
| 17 | R | R | R |
| 18 | R | R | R |
| 19 | R | R | R |
| 20 | R | R | R |
| 21 | R | R | R |
| 22 | R | R | R |
| 23 | R | R | R |
| 24 | R | R | R |
| 25 | R | R | R |
| 26 | R | R | S |
| 27 | R | R | R |
| 28 | R | R | R |
| 29 | R | R | R |
| 30 | R | R | R |
| 31 | R | R | R |
| 32 | R | R | S |
| 33 | R | R | R |
| 34 | R | R | R |
| 35 | R | R | R |
| fluoroquinolone sensitive strains |  |  |  |
| 1 | S | S | S |
| 2 | S | S | S |
| 3 | S | S | S |
| 4 | S | S | S |
| 5 | S | S | S |
| 6 | S | S | S |
| 7 | S | S | S |
| 8 | S | S | S |
| 9 | S | S | S |
| 10 | S | S | S |
| 11 | S | S | S |
| 12 | S | S | S |
| 13 | S | S | S |
| 14 | S | S | S |
| 15 | S | S | S |
| 16 | S | S | S |
| 17 | S | S | S |
| 18 | S | S | S |
| 19 | S | S | S |
| 20 | S | S | S |
| 21 | S | S | S |
| 22 | S | S | S |
| 23 | S | S | S |
| 24 | S | S | S |
| 25 | S | S | S |
| 26 | S | S | S |
| 27 | S | S | S |
| 28 | S | S | S |
| 29 | S | S | S |
| 30 | S | S | S |
| 31 | S | S | S |
| 32 | S | S | S |
| 33 | S | S | S |
| 34 | S | S | S |
| 35 | S | S | S |
| 36 | S | S | S |
| 37 | S | S | S |
| 38 | S | S | S |
| 39 | S | S | S |
| 40 | S | S | S |

OFX, ofloxacin; LFX, levofloxacin; MFX, moxifloxacin. R, resistance; S, sensitive.
